# Analytic Approximation for Bachelier Option Prices and Applications

**DOI:** 10.3390/e28060642

**Published:** 2026-06-06

**Authors:** Elisa Alòs, Òscar Burés

**Affiliations:** 1 Department of Economics and Business, Barcelona School of Economics, Universitat Pompeu Fabra, Ramón Trias Fargas 25-27, 08005 Barcelona, Spain; 2 Departament de Matemàtica Econòmica, Financera i Actuarial, Universitat de Barcelona, Diagonal 690–696, 08034 Barcelona, Spain; oscar.bures@ub.edu

**Keywords:** Bachelier-type model, option price expansion, Monte Carlo prices

## Abstract

It is well-known that, in the Bachelier model, when asset prices and volatilities are uncorrelated, the at-the-money implied volatility coincides with the fair value of the volatility swap. Using this identity as a starting point and applying classical Itô calculus and Taylor expansions, we write the price for out-of the-money (OTM) and in-the-money (ITM) options as an expansion with respect to the moneyness, where the coefficients are related to the negative (non-integer) powers of the future mean volatility. As an a application, we use it as a control variate to reduce the variance of Monte Carlo option prices in the correlated case.

## 1. Introduction

Today, option pricing theory is based largely on the Black–Scholes model, in which asset prices are log-normal. Most of the popular models in the financial industry (such as local or stochastic volatility models) are extensions of it. In this framework, asset prices are positive. This hypothesis is not always satisfied (as has recently been registered for commodities). In some scenarios, markets have moved to the Bachelier model (see [1,2]), where asset prices are assumed to be normal.

One of the main problems in option pricing (both in the Black–Scholes and in the Bachelier framework) is the construction of adequate closed-form approximation formulas for option prices and implied volatilities. To this end, several works are devoted to constructing expansions in which the leading term is the Black–Scholes/Bachelier price evaluated as a proxy for the implied volatility, which is usually the spot volatility or the variance swap. One classical approach relies on the analysis of the corresponding PDE with respect to a specific model parameter (see, among others [3,4,5,6]). Other researchers follow a probabilistic approach, where option prices depend on the joint distribution of the variance swap and asset prices (see, for example [7,8,9,10,11]). The results obtained in these latest works are very general and can be applied when the volatility is not Markovian, as in the case of rough volatilities. Some specific works on the Bachelier implied volatility include [12,13,14] and the references therein.

In all the above papers, the expansion contains a first correction term due to the correlation (associated with the leverage swap), a second one due to the vol-of-vol (associated with the quadratic variation of the variance swap), and higher-order terms. Even when these approximations work well near at-the-money strikes, they are not analytical (see, for example [15]), and their region of validity is limited.

Our purpose in this paper is to obtain an analytical expansion for Bachelier option prices in the case of uncorrelated asset prices and volatilities. Through adequate decomposition formulas, we write the option price as the ATM price plus a correction due to the moneyness. Then, a Taylor expansion allows us to write this correction in terms of powers of the moneyness, with coefficients depending on negative (non-integer) powers of the future integrated volatility.

Our numerical examples on the SABR and the Heston model confirm the validity of this approximation. As an application, we use it as a control variate in the simulation of option prices. This technique leads to a significant variance reduction in the Monte Carlo option pricing.

## 2. Preliminaries

We consider the Bachelier-type model for asset prices under a risk-neutral probability *P*:

(1) $$dX_{t}=\sigma_{t}\left(\rho dW_{t}+\sqrt{1-\rho^{2}}B_{t}\right),\;t\in \left[0,T\right]$$

for some T>0, where *W* and *B* are independent standard Brownian motions, $\rho \in \left[-1,1\right],$ and σ is a square integrable process adapted to the filtration generated by the Brownian motion *W*. As in the previous chapters, we denote by $F^{W}$ and $F^{B}$ the filtrations generated by *W* and *B*, respectively, and $F:=F^{W}\vee F^{B}$. If σ is constant and ρ=0, the above model is called the **Bachelier model**.

We denote by Bac(T,x,k,σ) the classical Bachelier price of a European call with time to maturity *T*, current stock price *x*, strike price *k* and volatility σ. That is,

$$Bac\left(T,x,k,\sigma \right)=\left(x-k\right)N\left(d_{Bac}\left(k,\sigma \right)\right)+N^{\prime }\left(d_{Bac}\left(k,\sigma \right)\right)\sigma \sqrt{T},$$

with

$$d_{Bac}\left(k,\sigma \right)=\frac{x-k}{\sigma \sqrt{T}},$$

where *N* is the cumulative distribution function and $N^{\prime }$ is the probability density function of the standard normal random variable.

We denote by $L_{Bac}\left(\sigma \right)$ the Bachelier differential operator with volatility σ:

$$L_{Bac}\left(\sigma \right)=\frac{\partial }{\partial t}+\frac{1}{2}\sigma^{2}\frac{\partial^{2}}{\partial x^{2}}$$

It is well known that $L_{Bac}\left(\sigma \right)Bac\left(\cdot ,\cdot ,\cdot ;\sigma \right)=0.$

Finally, we define the Bachelier implied volatility of a traded call option $I^{Bac}\left(k\right)$ as the unique volatility parameter one should put in the Bachelier formula to get the market option price *V*. That is, the quantity $I^{Bac}\left(k\right)$ such that

$$V=Bac(T,X_{0},k,I^{Bac}\left(k\right)),$$

where $X_{0}$ denotes the asset price and *k* the strike price of the option. Notice that, if $k=X_{0}$,

(2) $$V=Bac\left(T,X_{0},X_{0},I^{Bac}\left(X_{0}\right)\right)=N^{\prime }\left(0\right)I^{Bac}\sqrt{T}=\frac{1}{\sqrt{2\pi }}I^{Bac}\left(X_{0}\right)\sqrt{T}.$$

At the same time, due to the definition of the Black–Scholes implied volatility *I*,

(3) $$V=BS\left(T,X_{0},X_{0},I\left(X_{0}\right)\right)=X_{0}\left(2N\left(\frac{I\left(X_{0}\right)\sqrt{T}}{2}\right)-1\right),$$

where

$$BS\left(T,X_{0},k,\sigma \right)=e^{X_{0}}\Phi \left(d_{+}\right)-e^{k}\Phi \left(d_{-}\right),\;d_{\pm }=\frac{X_{0}-k}{\sigma \sqrt{T}}\pm \frac{\sigma }{2}\sqrt{T}.$$

Then, (2) and (3) imply the following conversion formula for ATM implied volatilities:

(4) $$I^{Bac}\left(X_{0}\right)=\frac{\sqrt{2\pi }}{\sqrt{T}}X_{0}\left(2N\left(\frac{I\left(X_{0}\right)\sqrt{T}}{2}\right)-1\right).$$

We will also need the following notations.

- $v=\sqrt{\frac{1}{T}E\int_{0}^{T}\sigma_{s}^{2}ds}$ is the square root of the variance swap.
- $\hat{v}=E\sqrt{\frac{1}{T}\int_{0}^{T}\sigma_{s}^{2}ds}$ is the volatility swap.
- For all s∈[0,T], we define $M_{s}=\frac{1}{T}E_{s}\int_{0}^{T}\sigma_{u}^{2}du$.
- For all s∈[0,T], we denote $v_{s}=\sqrt{\frac{1}{T}E_{s}\int_{0}^{T}\sigma_{u}^{2}du}$. In particular, $v_{0}=v.$

Notice that $v=\sqrt{M_{0}}$, $v_{s}=\sqrt{M_{s}}$, and $\hat{v}=E\sqrt{M_{T}}$. Then, a direct application of Itô’s formula to the process *M* and the function $f\left(x\right)=\sqrt{x}$ leads to the following relationship between the variance and the volatility swap

(5) $$\hat{v}=v-\frac{1}{8}E\int_{0}^{T}\frac{1}{v_{s}^{3}}d{\langle M,M\rangle }_{s}$$

## 3. An Analytical Expansion for Option Prices

Our approach is based on the following decomposition for option prices in the uncorrelated case. We assume the following integrability condition.

(H) For all p>1, $v^{-1}$ and $|\frac{d{\langle M,M\rangle }_{s}}{ds}|$ are in $L^{p}\left(\left[0,T\right]\times \Omega \right)$.

**Proposition** **1** (Decomposition formula for option prices in the uncorrelated case). *Consider the model (1) with ρ=0 and assume that Hypothesis (H) holds. Then*

$$\begin{array}{ccc} V & = & Bac(T,X_{0},k,v)\\ & & +\frac{T^{2}}{8}\;E\left(\int_{0}^{T}K_{Bac}\left(T,X_{0},k,v_{s}\right)d{\langle M,M\rangle }_{s}\right), \end{array}$$

*where*

$$\begin{array}{ccc} K_{Bac}\left(T,x,\sigma \right) & = & \frac{\partial^{4}Bac}{\partial x^{4}}\left(T,x,\sigma \right)\\ & = & \frac{{\left(x-k\right)}^{2}-T\sigma^{2}}{T^{\frac{5}{2}}\sigma^{5}}\frac{\exp\left(-\frac{d_{Bac}^{2}\left(\sigma \right)}{2}\right)}{\sqrt{2\pi }} \end{array}$$

**Proof.**  Since the value of the option at the time to maturity coincides with the payoff, we can condition on the volatility paths and take expectation to write the option price *V* as

$$V=E(Bac\left(T,X_{0},k,v_{T}\right))$$

Now, a direct application of Itô’s formula and the fact that

$$\frac{\partial Bac}{\partial \sigma }\left(T,X_{0},k,\sigma \right)\frac{1}{\sigma T}=\frac{\partial^{2}Bac}{\partial x^{2}}\left(T,X_{0},k,\sigma \right)$$

give us that

$$\begin{array}{ccc} Bac(T,X_{0},k,v_{T}) & = & Bac(T,X_{0},k,v_{T})\\ & + & \frac{1}{2}T\int_{0}^{T}\frac{\partial^{2}Bac}{\partial x^{2}}\left(T,X_{0},k,v_{s}\right)dM_{s}\\ & + & \frac{1}{8}T^{2}\int_{0}^{T}\frac{\partial^{4}Bac}{\partial x^{4}}\left(T,X_{0},k,v_{s}\right)d{\langle M,M\rangle }_{s}. \end{array}$$

Then, taking expectations, and taking into account that $v_{T}=v$, we get

$$\begin{array}{ccc} V & = & Bac(T,X_{0},k,v)\\ & + & \frac{1}{8}T^{2}E\int_{0}^{T}\frac{\partial^{4}Bac}{\partial x^{4}}\left(T,X_{0},k,v_{s}\right)d{\langle M,M\rangle }_{s}, \end{array}$$

and now the proof is complete. □

As a direct corollary, we get the following decomposition formula

**Corollary** **1.** *Consider the model (1) and assume that hypothesis (H) holds. Then*

$$\begin{array}{ccc} V=Bac(T,X_{0},k,v) & + & \frac{{\left(X_{0}-k\right)}^{2}}{8T^{\frac{1}{2}}\sqrt{2\pi }}E\int_{0}^{T}\frac{1}{v_{s}^{5}}\exp\left(-\frac{d_{Bac}^{2}\left(v_{s}\right)}{2}\right)d{\langle M,M\rangle }_{s}\\ & - & \frac{1}{8}\frac{\sqrt{T}}{\sqrt{2\pi }}E\left(\int_{0}^{T}\exp\left(-\frac{d_{Bac}^{2}\left(\sigma \right)}{2}\right)\frac{1}{v_{s}^{3}}d{\langle M,M\rangle }_{s}\right). \end{array}$$

**Proof.**  Notice that

$$\begin{array}{ccc} K_{Bac}\left(T,x,k,\sigma \right) & = & \frac{{\left(x-k\right)}^{2}-T\sigma^{2}}{T^{\frac{5}{2}}\sigma^{5}}\frac{\exp\left(-\frac{d_{Bac}^{2}\left(\sigma \right)}{2}\right)}{\sqrt{2\pi }}\\ & = & \frac{{\left(x-k\right)}^{2}}{T^{\frac{5}{2}}\sigma^{5}}\frac{\exp\left(-\frac{d_{Bac}^{2}\left(\sigma \right)}{2}\right)}{\sqrt{2\pi }}\\ & - & \frac{1}{T^{\frac{3}{2}}\sigma^{3}\sqrt{2\pi }}\exp\left(-\frac{d_{Bac}^{2}\left(\sigma \right)}{2}\right). \end{array}$$

Now, as

$$\frac{\partial Bac}{\partial \sigma }\left(T,x,k,v_{s}\right)=\frac{\sqrt{T}}{\sqrt{2\pi }}\exp\left(-\frac{d_{Bac}^{2}\left(\sigma \right)}{2}\right)$$

it follows that

$$\begin{array}{ccc} K_{Bac}\left(T,x,k,\sigma \right) & = & \frac{{\left(x-k\right)}^{2}}{T^{\frac{5}{2}}\sigma^{5}}\frac{\exp\left(-\frac{d_{Bac}^{2}\left(\sigma \right)}{2}\right)}{\sqrt{2\pi }}\\ & - & \frac{1}{T^{2}\sigma^{3}}\frac{\partial Bac}{\partial \sigma }\left(T,x,k,v_{s}\right). \end{array}$$

Then, Proposition 1 leads to

$$\begin{array}{ccc} V=Bac(T,X_{0},k,v) & + & \frac{{\left(X_{0}-k\right)}^{2}}{8T^{\frac{1}{2}}\sqrt{2\pi }}E\int_{0}^{T}\frac{1}{v_{s}^{5}}\exp\left(-\frac{d_{Bac}^{2}\left(v_{s}\right)}{2}\right)d{\langle M,M\rangle }_{s}\\ & - & \frac{1}{8}\frac{\sqrt{T}}{\sqrt{2\pi }}E\left(\int_{0}^{T}\exp\left(-\frac{d_{Bac}^{2}\left(\sigma \right)}{2}\right)\frac{1}{v_{s}^{3}}d{\langle M,M\rangle }_{s}\right). \end{array}$$

□

**Remark** **1** (The ATMI and the volatility swap). *Notice that, if k=x, $\frac{\partial Bac}{\partial \sigma }\left(T,x,k,v_{s}\right)$ is deterministic and then*

$$Bac\left(T,X_{0},X_{0},\hat{v}\right)=Bac\left(T,X_{0},X_{0},v\right)-\frac{1}{8}\;E\left(\int_{0}^{T}\frac{\partial Bac}{\partial \sigma }\left(T,X_{0},X_{0},v_{s}\right)\frac{1}{v_{s}^{3}}d{\langle M,M\rangle }_{s}\right),$$

*which implies that, for ATM options, $V=Bac(T,X_{0},X_{0},\hat{v})$, according to the well-known properties of the Bachelier implied volatility.*

Now we are in a position to prove the main result of this paper.

**Theorem** **1** (Price expansion). *Consider the model (1) with ρ=0 and assume that Hypothesis (H) holds. Then*

(6) $$\begin{array}{ccc} V & = & Bac\left(T,X_{0},k,v\right)+\frac{T^{\frac{1}{2}}}{\sqrt{2\pi }}\left(\hat{v}-v\right)\\ & - & \frac{T^{\frac{1}{2}}}{2\sqrt{2\pi }}\sum_{n=1}\frac{1}{n!(2n-1)}{\left(-\frac{{\left(X_{0}-k\right)}^{2}}{2T}\right)}^{n}\\ & & \times \left[E{\left(\frac{1}{T}\int_{0}^{T}\sigma_{s}^{2}ds\right)}^{\frac{1}{2}-n}-{\left(\frac{1}{T}\int_{0}^{T}E\left(\sigma_{s}^{2}\right)ds\right)}^{\frac{1}{2}-n}\right] \end{array}$$

*provided the right hand side is convergent.*

**Proof.**  A Taylor expansion of the exponential functions in Corollary 1 gives

$$\begin{array}{ccc} V=Bac(T,X_{0},k,v) & - & \frac{T^{\frac{1}{2}}}{4\sqrt{2\pi }}E\int_{0}^{T}\frac{1}{v_{s}^{3}}\left(-\frac{{\left(X_{0}-k\right)}^{2}}{2v_{s}^{2}T}\right)\exp\left(-\frac{{\left(X_{0}-k\right)}^{2}}{2v_{s}^{2}T}\right)d{\langle M,M\rangle }_{s}\\ & - & \frac{1}{8}\frac{\sqrt{T}}{\sqrt{2\pi }}E\left(\int_{0}^{T}\exp\left(-\frac{{\left(X_{0}-k\right)}^{2}}{2v_{s}^{2}T}\right)\frac{1}{v_{s}^{3}}d{\langle M,M\rangle }_{s}\right)\\ =Bac(T,X_{0},k,v) & - & \frac{T^{\frac{1}{2}}}{4\sqrt{2\pi }}\sum_{n=1}\frac{1}{(n-1)!}\left[{\left(-\frac{{\left(X_{0}-k\right)}^{2}}{2T}\right)}^{n}E\int_{0}^{T}\frac{1}{v_{s}^{3+2n}}d{\langle M,M\rangle }_{s}\right]\\ & - & \frac{1}{8}\frac{T^{\frac{1}{2}}}{\sqrt{2\pi }}\sum_{n=0}\frac{1}{n!}\left[{\left(-\frac{{\left(X_{0}-k\right)}^{2}}{2v_{s}^{2}T}\right)}^{n}E\int_{0}^{T}\frac{1}{v_{s}^{3+2n}}d{\langle M,M\rangle }_{s}\right]\\ =Bac(T,X_{0},k,v) & - & \frac{1}{8}\frac{T^{\frac{1}{2}}}{\sqrt{2\pi }}E\int_{0}^{T}\frac{1}{v_{s}^{3}}d{\langle M,M\rangle }_{s}\\ & - & \frac{T^{\frac{1}{2}}}{\sqrt{2\pi }}\sum_{n=1}\left(\frac{1}{4(n-1)!}+\frac{1}{8n!}\right)\left[{\left(-\frac{{\left(X_{0}-k\right)}^{2}}{2T}\right)}^{n}E\int_{0}^{T}\frac{1}{v_{s}^{3+2n}}d{\langle M,M\rangle }_{s}\right]\\ =Bac(T,X_{0},k,v) & - & \frac{1}{8}\frac{T^{\frac{1}{2}}}{\sqrt{2\pi }}E\int_{0}^{T}\frac{1}{v_{s}^{3}}d{\langle M,M\rangle }_{s}\\ & - & \frac{T^{\frac{1}{2}}}{\sqrt{2\pi }}\sum_{n=1}\frac{2n+1}{8n!}\left[{\left(-\frac{{\left(X_{0}-k\right)}^{2}}{2T}\right)}^{n}E\int_{0}^{T}\frac{1}{v_{s}^{3+2n}}d{\langle M,M\rangle }_{s}\right]. \end{array}$$

Now, notice that, for all real θ

$$E\left(M_{T}^{\theta /2}\right)=M_{0}^{\theta /2}+\frac{1}{2}\frac{\theta }{2}\left(\frac{\theta }{2}-1\right)E\int_{0}^{T}v_{s}^{\left(\theta -4\right)}d{\langle M,M\rangle }_{s}.$$

Taking −3−2n=θ−4 we have θ=1−2n and then $\frac{\theta }{2}\left(\frac{\theta }{2}-1\right)=n^{2}-0.25$. This implies that

(7) $$\begin{array}{ccc} E\int_{0}^{T}\frac{1}{v_{s}^{3+2n}}d{\langle M,M\rangle }_{s} & = & \frac{2}{n^{2}-0.25}\left(E\left(M_{T}^{1/2-n}\right)-M_{0}^{1/2-n}\right)\\ & = & \frac{2}{n^{2}-0.25}\left(E{\left(\frac{1}{T}\int_{0}^{T}\sigma_{s}^{2}ds\right)}^{\frac{1}{2}-n}-{\left(\frac{1}{T}\int_{0}^{T}E\sigma_{s}^{2}ds\right)}^{\frac{1}{2}-n}\right), \end{array}$$

and now the proof is complete. □

**Remark** **2.** *The convergence of the series given in Theorem 1 needs to be checked model by model. For instance, in the SABR model, one can prove by means of the Hartman–Watson distribution that the series is convergent if $|X_{0}-k|<\frac{\sigma_{0}}{\nu }$, where ν denotes the vol-of-vol. If convergent, this result reduces the computation of option prices $Bac(T,X_{0},k,v_{T})$ to the estimation of the corresponding negative (non-integer) moments of $M_{T}$. Once these moments are obtained and stored, the calculation of the option price in any concrete strike k is obtained via a closed-form formula.*

**Remark** **3.** *Theorem 1 does not only give an expression for the option price, but it also allows us to deduce, taking derivatives, an analytical formula for the Greeks. For example, the Delta *Δ* of a call is given by*

(8) $$\begin{array}{ccc} \Delta & = & N(d_{Bac}\left(v\right))\\ & + & \frac{\left(X_{0}-k\right)}{\sqrt{2T\pi }}\sum_{n=1}\frac{1}{\left(n-1)!(2n-1\right)}{\left(-\frac{{\left(X_{0}-k\right)}^{2}}{2T}\right)}^{n-1}\\ & & \times \left[E{\left(\frac{1}{T}\int_{0}^{T}\sigma_{s}^{2}ds\right)}^{\frac{1}{2}-n}-{\left(\frac{1}{T}\int_{0}^{T}E\left(\sigma_{s}^{2}\right)ds\right)}^{\frac{1}{2}-n}\right]. \end{array}$$

*and the following expression for the Gamma *Γ* holds:*

(9) $$\begin{array}{ccc} \Gamma & = & \frac{1}{\sqrt{2\pi T}v}e^{-d_{Bac}^{2}\left(v\right)/2}\\ & + & \frac{1}{\sqrt{2T\pi }}\sum_{n=1}\frac{1}{(n-1)!}{\left(-\frac{{\left(X_{0}-k\right)}^{2}}{2T}\right)}^{n-1}\\ & & \times \left[E{\left(\frac{1}{T}\int_{0}^{T}\sigma_{s}^{2}ds\right)}^{\frac{1}{2}-n}-{\left(\frac{1}{T}\int_{0}^{T}E\left(\sigma_{s}^{2}\right)ds\right)}^{\frac{1}{2}-n}\right]. \end{array}$$

**Remark** **4.** *An analytical expression for the Bachelier price in the uncorrelated case can be the starting point for several applications. In the next section, we will see how to use it as a control variate in the Monte Carlo computation of option prices in the correlated case.*

## 4. Numerical Examples

**Example** **1** (The Heston model )**.** *Let us assume a Heston–Bachelier model where the volatility process is given by*

(10) $$d\sigma_{t}^{2}=-\kappa \left(\sigma_{t}^{2}-\theta \right)dt+\nu \sqrt{\sigma_{t}^{2}}dB_{t},$$

*where κ,θ, and ν are positive real numbers. Then, a straightforward computation leads to*

$$M_{0}=\theta +\frac{\sigma^{2}-\theta }{\kappa T}\left(1-e^{-\kappa T}\right).$$

*Consider the parameters $\sigma_{0}=20,\kappa =2,\theta =400,$ and ν=20. The first thing we will explore is how well the approximation given by Theorem 1 works versus a benchmark. As a benchmark, we have chosen uncorrelated call prices with initial asset price $X_{0}=100$, maturities T∈{0.8,1.0,1.2} and strikes k∈[70,140]. The values of such options are computed with 100,000 conditional Monte Carlo simulations with antithetic variables. For the expansion, we have chosen N=30 as the number of terms. The moments*

$$E\left[{\left(\frac{1}{T}\int_{0}^{T}\sigma_{s}^{2}ds\right)}^{\frac{1}{2}-n}\right]$$

**Figure 1:**
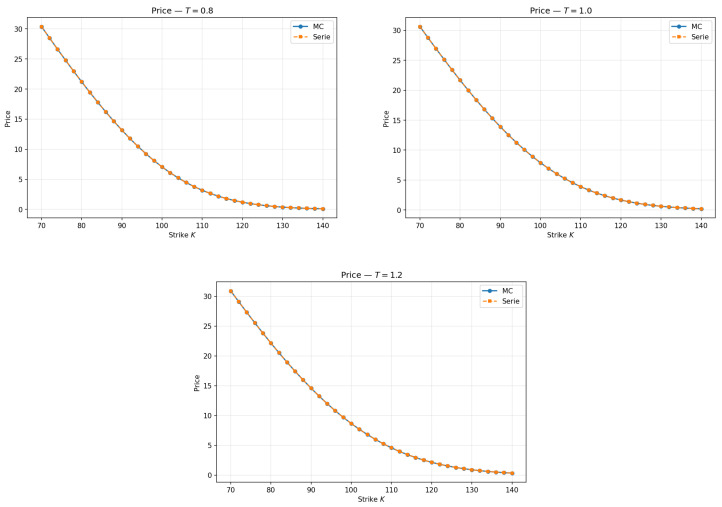
Approximation of option prices for the Heston model.

**Figure 2:**
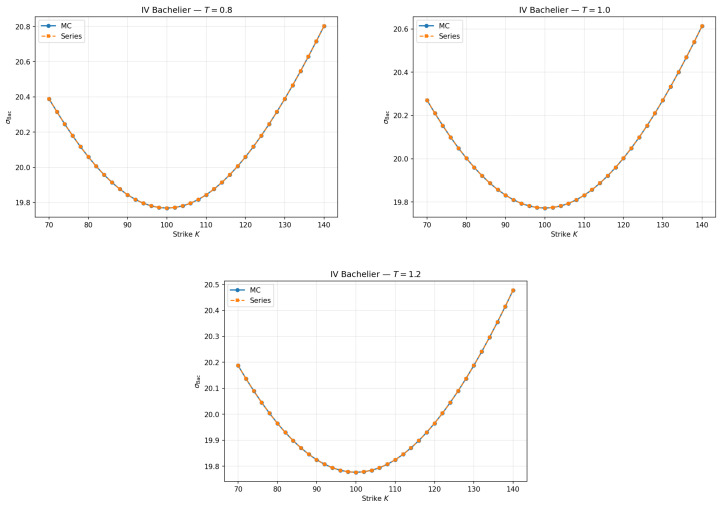
Approximation of implied volatilities for the Heston model.

*have been computed using Monte Carlo. In Figure 1, we see how our approximation accurately fits the option prices. In order to confirm the high accuracy of our approximation, in Figure 2 we see that the excellent option price fitting is also translated into a highly accurate fit of the implied volatility smiles.*

*Since the precision for N=30 is quite high, one may wonder how many terms are needed to obtain a certain level of accuracy. In order to answer this question, we have found the minimum number $N^{*}$ such that the error between the implied volatilites computed with $N^{*}$ and $N^{*}+1$ terms is less than 0.01. In a sense, $N^{*}$ denotes the term after which adding more terms does not substantially change the approximation of the implied volatility. In Table 1, Table 2 and Table 3 we detail such an “optimal” number of terms $N^{*}$ for a selection of the options used for the implied volatility fitting.*

**Example** **2** (The SABR model)**.** *Let us consider the SABR model where*

$$\sigma_{t}=\sigma_{0}\exp\left(-\frac{\nu^{2}}{2}t+\nu B_{t}\right).$$

*Then a direct computation leads to*

$$M_{0}=\sigma_{0}^{2}\left(\frac{\exp\nu^{2}T-1}{\nu^{2}T}\right).$$

**Figure 3:**
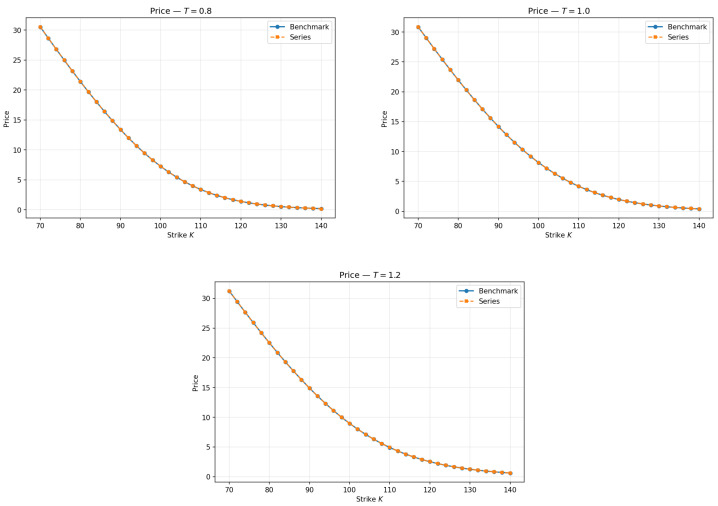
Approximation of option prices for the SABR model.

**Figure 4:**
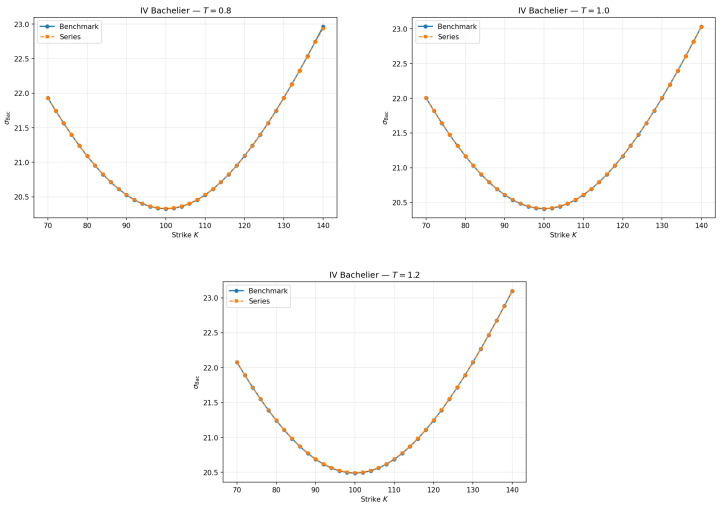
Approximation of implied volatilities for the SABR model.

*Consider the parameters $\sigma_{0}=20$, and ν=0.5. In the following plots, we can see the goodness of approximation of the series for option prices and implied volatilities. As a benchmark, we consider the prices computed as in Equation (3.91) of [16]. For the expansion we have taken N=30 terms. Again, the negative moments of the integrated variance are computed by Monte Carlo. Another possible approach, which is especially useful for short maturities, is to use the semi-analytical expression of the negative moments of the integrated variance of the SABR model by means of the Hartman–Watson distribution. For more information about the computation of these moments, see [17]. In Figure 3 and Figure 4 we can see how well our method fits the option prices and their implied volatilities.*

*As can be observed, our method closely matches the benchmark curve. To give an idea of the accuracy of our method, in Table 4, Table 5 and Table 6 we display the relative error between the benchmark and the approximated implied volatilities in order to show that the implied volatilities obtained by the call prices computed as in Theorem 1 provide an excellent fit.*

**Figure 5:**
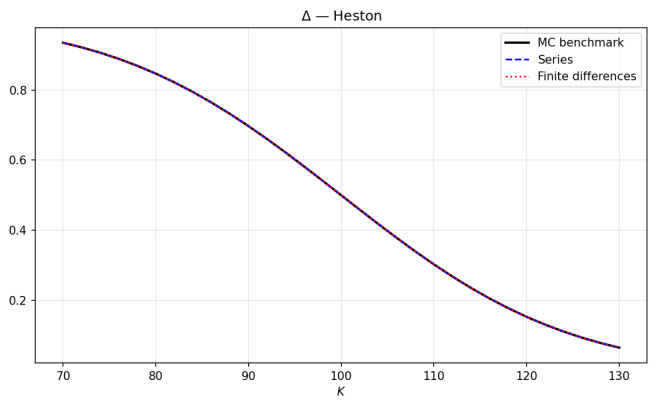
Greek Δ computed by the 3 stated methods with $X_{0}=100$ and T=1.0.

**Example** **3** (Computation of Greeks)**.** *As has been mentioned in Remark 3, differentiating the expression derived in Theorem 1 with respect to $X_{0}$ provides an analytical way to compute the *Δ* and the *Γ* of the options in a fast and accurate way. To show this, consider first the Heston model using the same set of parameters as in Example 4.1. We will compute the *Δ* of several options under the Bachelier Heston model with our method and we will compare it to the *Δ* obtained by finite differences with a step-size $h=10^{-3}$. The benchmark is the *Δ* computed by differentiating the conditional Monte Carlo expectation. As is seen in Figure 5, the three methods provide an excellent fit of the *Δ* of the option. The difference between our method and the other two is the computational cost. In Table 7, we see that our method is the fastest in the computation of the *Δ*.*

**Figure 6:**
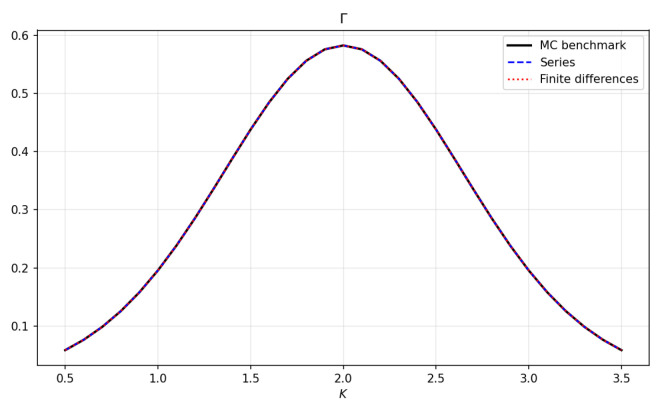
Greek Γ computed by the 3 stated methods with $X_{0}=2$ and T=1.0.

*A similar phenomenon happens with the computation of Gamma; in this case we consider the Bachelier SABR model with $X_{0}=2$, $\sigma_{0}=0.7$ and ν=0.3 for the sake of diversity. In Figure 6, we observe that again the fit provided by the three methods is excellent. Table 8 shows again that our method outperforms the other two in computational speed.*

**Example** **4** (Monte Carlo Variance Reduction)**.** *Another interesting quality of the expansion provided in Theorem 1 is that, for certain option, it works as an excellent control variate. To show the variance reduction, we will consider three different models used for computing options:*

***(I)***     ***Bachelier Heston*** *model with $X_{0}=100$, $\sigma_{0}=20$, κ=2, θ=400, ν=20 and ρ=−0.3.*
***(II)***    ***Bachelier SABR*** *model with $X_{0}=100$, $\sigma_{0}=20$, ν=0.5 and ρ=−0.5.*
***(III)***  ***Bachelier SABR*** *model with $X_{0}=2$, $\sigma_{0}=0.7$, ν=0.3 and ρ=−0.3.*

*As control variates, we will study the variance reduction of the following choices:*

***(CV1)***  
*A linear control variate $X_{T}-X_{0}$ where X follows one of the models **(I)**–**(III)***.
***(CV2)*** 
*A control variate based on the variance swap, that is,*
  $$\frac{1}{T}\int_{0}^{T}\sigma_{s}^{2}ds-E\left[\frac{1}{T}\int_{0}^{T}\sigma_{s}^{2}ds\right].$$
***(CV3)*** 
*A control variate based on the volatility swap, that is,*
  $$\sqrt{\frac{1}{T}\int_{0}^{T}\sigma_{s}^{2}ds}-E\left[\sqrt{\frac{1}{T}\int_{0}^{T}\sigma_{s}^{2}ds}\right].$$
***(CV4)*** 
*A control variate based on the expansion given in Theorem 1, that is,*
  $${\left(X_{T}^{0}-K\right)}_{+}-V,$$
  *where $X^{0}$ denotes one of the models* ***(I)*** *–* ***(III)*** *with ρ=0 and V is the price of the option under model $X^{0}$ computed via the expansion given in Theorem 1.*

*For every control variate Z selected between* ***(CV1)*** *–* ***(CV4)*** *we will find $\beta^{*}$ such that*

$$\beta^{*}=\arg\min_{\beta }\left({\left(X_{T}-K\right)}_{+}-\beta Z\right).$$

*In order to highlight the variance reduction, we will plot the following two quantities:*

$$\left({\left(X_{T}-K\right)}_{+}-\beta^{*}Z\right),\;\frac{\left({\left(X_{T}-K\right)}_{+}\right)}{\left({\left(X_{T}-K\right)}_{+}-\beta^{*}Z\right)}.$$

**Figure 7:**
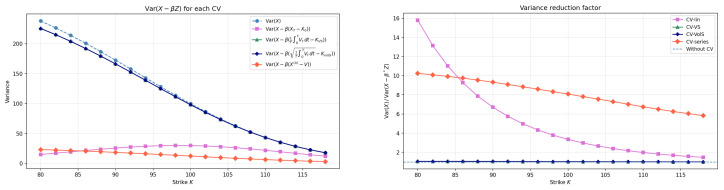
Variance and variance reduction factor for each control variate in model **(I)**.

**Figure 8:**
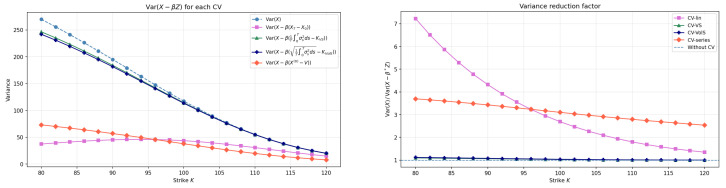
Variance and variance reduction factor for each control variate in model **(II)**.

**Figure 9:**
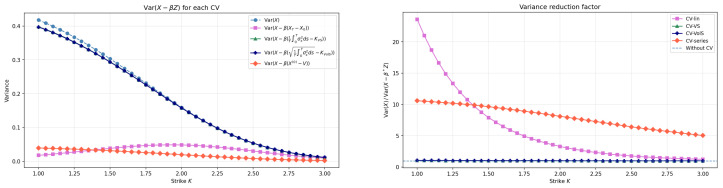
Variance and variance reduction factor for each control variate in model **(III)**.

*In Figure 7, Figure 8 and Figure 9 we see that our control variate,* ***(CV4)*** *, outperforms the other control variates in the OTM regime. Near ATM our control variate works better when ρ=−0.3. In fact, it is expected that the performance of our control variate decreases as |ρ|→1. In the deep ITM regime, since the payoff satisfies ${\left(X_{T}-K\right)}_{+}\approx X_{T}-K$, it is natural that the linear control variate $X_{T}-X_{0}$ is the one that exhibits the most variance reduction.*

## 5. Discussion

We have obtained an analytical expansion for Bachelier–stochastic volatility models in the case of uncorrelated asset prices and volatilities. Our expansion, based on the Itô calculus and Taylor expansions, allows us to compute option prices with a reduced number of computations. As an application, we also derive approximations for the Delta and the Gamma of the options. Moreover, we obtain a strong variance reduction if we use this approximation as a control variate for pricing options where the volatility and the asset price are correlated.

## Figures and Tables

**Table 1:** Optimal number of terms and error for T=0.8.

Strike	$N^{*}$	Error with Next Term
70	9	0.004039
78	5	0.004954
86	3	0.001626
94	1	0.002159
102	1	0.000025
110	2	0.001593
118	4	0.002405
126	7	0.003052
134	12	0.004557

**Table 2:** Optimal number of terms and error for T=1.0.

Strike	$N^{*}$	Error with Next Term
70	7	0.005036
78	4	0.006216
86	2	0.006591
94	1	0.001379
102	1	0.000016
110	2	0.000755
118	3	0.005396
126	5	0.009715
134	9	0.005078

**Table 3:** Optimal number of terms and error for T=1.2.

Strike	$N^{*}$	Error with Next Term
70	6	0.004298
78	4	0.002175
86	2	0.003477
94	1	0.000919
102	1	0.000011
110	1	0.007597
118	3	0.002329
126	5	0.002575
134	7	0.008735

**Table 4:** Relative error in implied volatility for T=0.8.

Strike	Rel. Error	Strike	Rel. Error
70	1.487 $\times \;10^{-4}$	106	$1.462\times 10^{-4}$
72	$1.176\times 10^{-4}$	108	$1.326\times 10^{-4}$
74	$8.723\times 10^{-5}$	110	$1.159\times 10^{-4}$
76	$5.770\times 10^{-5}$	112	$9.638\times 10^{-5}$
78	$2.901\times 10^{-5}$	114	$7.454\times 10^{-5}$
80	$1.268\times 10^{-6}$	116	$5.076\times 10^{-5}$
82	$2.539\times 10^{-5}$	118	$2.539\times 10^{-5}$
84	$5.076\times 10^{-5}$	120	$1.268\times 10^{-6}$
86	$7.454\times 10^{-5}$	122	$2.901\times 10^{-5}$
88	$9.638\times 10^{-5}$	124	$5.770\times 10^{-5}$
90	$1.159\times 10^{-4}$	126	$8.723\times 10^{-5}$
92	$1.326\times 10^{-4}$	128	$1.176\times 10^{-4}$
94	$1.462\times 10^{-4}$	130	$1.487\times 10^{-4}$
96	$1.562\times 10^{-4}$	132	$1.806\times 10^{-4}$
98	$1.623\times 10^{-4}$	134	$2.132\times 10^{-4}$
100	$1.644\times 10^{-4}$	136	$2.477\times 10^{-4}$
102	$1.623\times 10^{-4}$	138	$3.181\times 10^{-4}$
104	$1.562\times 10^{-4}$	140	$1.328\times 10^{-3}$

**Table 5:** Relative error in implied volatility for T=1.0.

Strike	Rel. Error	Strike	Rel. Error
70	$1.072\times 10^{-4}$	106	$1.509\times 10^{-4}$
72	$8.478\times 10^{-5}$	108	$1.379\times 10^{-4}$
74	$6.138\times 10^{-5}$	110	$1.218\times 10^{-4}$
76	$3.727\times 10^{-5}$	112	$1.032\times 10^{-4}$
78	$1.274\times 10^{-5}$	114	$8.234\times 10^{-5}$
80	$1.190\times 10^{-5}$	116	$5.988\times 10^{-5}$
82	$3.624\times 10^{-5}$	118	$3.624\times 10^{-5}$
84	$5.988\times 10^{-5}$	120	$1.190\times 10^{-5}$
86	$8.234\times 10^{-5}$	122	$1.274\times 10^{-5}$
88	$1.032\times 10^{-4}$	124	$3.727\times 10^{-5}$
90	$1.218\times 10^{-4}$	126	$6.138\times 10^{-5}$
92	$1.379\times 10^{-4}$	128	$8.478\times 10^{-5}$
94	$1.509\times 10^{-4}$	130	$1.072\times 10^{-4}$
96	$1.605\times 10^{-4}$	132	$1.286\times 10^{-4}$
98	$1.663\times 10^{-4}$	134	$1.486\times 10^{-4}$
100	$1.683\times 10^{-4}$	136	$1.675\times 10^{-4}$
102	$1.663\times 10^{-4}$	138	$1.873\times 10^{-4}$
104	$1.605\times 10^{-4}$	140	$2.658\times 10^{-4}$

**Table 6:** Relative error in implied volatility for T=1.2.

Strike	Rel. Error	Strike	Rel. Error
70	$1.077\times 10^{-5}$	106	$2.132\times 10^{-4}$
72	$1.143\times 10^{-5}$	108	$2.025\times 10^{-4}$
74	$3.343\times 10^{-5}$	110	$1.892\times 10^{-4}$
76	$5.520\times 10^{-5}$	112	$1.738\times 10^{-4}$
78	$7.669\times 10^{-5}$	114	$1.566\times 10^{-4}$
80	$9.776\times 10^{-5}$	116	$1.380\times 10^{-4}$
82	$1.183\times 10^{-4}$	118	$1.183\times 10^{-4}$
84	$1.380\times 10^{-4}$	120	$9.776\times 10^{-5}$
86	$1.566\times 10^{-4}$	122	$7.669\times 10^{-5}$
88	$1.738\times 10^{-4}$	124	$5.520\times 10^{-5}$
90	$1.892\times 10^{-4}$	126	$3.343\times 10^{-5}$
92	$2.025\times 10^{-4}$	128	$1.143\times 10^{-5}$
94	$2.132\times 10^{-4}$	130	$1.077\times 10^{-5}$
96	$2.212\times 10^{-4}$	132	$3.317\times 10^{-5}$
98	$2.261\times 10^{-4}$	134	$5.580\times 10^{-5}$
100	$2.277\times 10^{-4}$	136	$7.874\times 10^{-5}$
102	$2.261\times 10^{-4}$	138	$1.025\times 10^{-4}$
104	$2.212\times 10^{-4}$	140	$1.379\times 10^{-4}$

**Table 7:** Computation times for the different methods.

Method	Time (s)
Benchmark MC	4.28
**Series**	**0.76**
Finite Differences	46.42

**Table 8:** Computation times for the different methods.

Method	Time (s)
Benchmark MC	4.38
**Series**	**0.95**
Finite Differences	4.65

## Data Availability

Codes available upon request.
